# A Prospective Observational Clinical and Radiological Study of a Modular Bridging External Fixator for Unstable Distal Radius Fractures

**DOI:** 10.5704/MOJ.2111.016

**Published:** 2021-11

**Authors:** G Maccagnano, G Noia, G Vicenti, M Coviello, V Pesce, B Moretti

**Affiliations:** 1 Department of Clinical and Experimental Medicine, Azienda Ospedaliero Universitaria Ospedali Riuniti - Foggia, Foggia, Italy; 2 Department of Basic Medical Science, Neuroscience and Sensory Organs, Azienda Ospedaliero Universitaria Consorziale Policlinico, Bari, Italy

**Keywords:** unstable distal radius fracture, modular external fixator, radius fracture, closed reduction and internal fixation (CRIF), wrist fracture

## Abstract

**Introduction:**

Distal Radius Fractures (DRFs), with a reported annual incidence of 600,000, are common injuries treated by trauma surgeons. This prospective observational study aims to assess the efficacy of a modular external fixation system in the treatment of unstable distal radius fractures at 12-months follow-up.

**Materials and methods:**

Between December 2014 and December 2016, 35 patients (female: 21, male:14; mean age: 62.5), with unstable DRFs, treated with modular external fixation system, were selected for this prospective observational study. All the patients underwent clinical and radiological reviews at follow-up.

**Results:**

At 12-month follow-up, a mean DASH score of 15.73 and a mean PRWE score 20.10 were recorded. Mean radial inclination was 19.92°; mean ulnar variance was 1.12 mm and mean palmar inclination was 9.76°.

**Conclusion:**

Modular external fixator system revealed clinically and radiologically effective in the treatment of unstable and comminuted DRFs. Additional K-wires should be used to complement the fracture fixation, when there is unacceptable fragment reduction only with external fixator.

## Introduction

Distal Radius Fractures (DRFs), with a reported annual incidence of 600,000, represent one of the common injuries treated by trauma surgeons^1^. These fractures tend to occur in a bimodal age distribution, in young patients involved in high-energy trauma and in elderly women after low-energy falls, secondary to osteoporosis^2^. Fracture comminution and articular involvement are more likely to be found in high-energy trauma^2-3^.

Indications for a correct treatment of DRFs (surgical or non-surgical) have evolved over time. However, several controversies remain about the management of DRFs^4-6^. None of the twenty-nine recommendations of the American Academy of Orthopaedic Surgeons (AAOS) for distal radius practice guidelines were graded as strong^7^. Moreover, the Cochrane Database has shown that evidence is lacking regarding many aspects of the treatment of DRFs^4,8^.

The optimal treatment of unstable DRFs is still a topic of debate and there is considerable disagreement about the need for strict anatomical restoration of the joint surface^4,9^. External fixation (EF) is a valid and simple method used for fixing unstable DRFs. A recent meta-analysis has shown that there were no significant different clinical outcomes in AO/OTA C-type managed with locking plates, compared with EF^10^. Modular external bridging fixator has replaced the previous fixed bridging, ensuring rapid mobilisation^11^.

This prospective observational study aims to assess the efficacy of a recent bridging modular external fixation system in the treatment of unstable distal radius fractures at 12 months follow-up.

## Materials and Methods

The present prospective observational study was started in December 2017. All patients with unstable DRFs, treated with modular external fixation system at our institution until December 2019 were potentially eligible for this study. Ethical approval was obtained from our centre’s clinical research ethics centre.

The inclusion criteria were: (a) age of patient above 18 years; (b) presence of unstable DRFs; (c) maximum 14 days post-trauma interval; (d) high-energy or low-energy trauma. Exclusion criteria were: (a) bilateral wrist fracture; (b) concomitant carpal bones fracture; (c) history of rheumatoid arthritis or wrist arthrodesis; (d) contraindication to EF.

The DRFs instability was defined as dorsal/volar comminution ratio >50%; dorsal/volar angle >20°; radial shortening >5-10mm; articular step-off >1mm; associated ulnar fracture^12-14^.

All the patients underwent wrist radiographs, in anteroposterior and lateral views, at the outset, after surgery, at time of mobilisation, at external fixator removal and at 12 months follow-up. The radial inclination and ulnar variance were assessed in the anteroposterior views, and palmar inclination was evaluated in the lateral view. The radiological evaluation was performed independently by two orthopaedic surgeons with more than five years of experience in the field of upper limb traumatology.

Clinical evaluation was performed, at baseline and at each follow-up visit, using the Disability of the Arm, Shoulder and Hand Score (DASH) and Patient-Related Wrist Evaluation (PRWE). The Range of Movement (ROM) and grip strength were also evaluated at each follow-up.

All the surgical procedures were performed by the same surgical team. Fracture reduction was obtained by external manipulation, under C-arm control. The fracture was then stabilised using a modular external fixation system [Galaxy Wrist, Orthofix, Texas, USA], in bridge configuration.

The two proximal screws were inserted in the middle third of the radial diaphysis located in the mediolateral position, taking care to avoid the brachioradialis tendon and the superficial radial nerve, or in the dorsolateral position, taking care to retract the long radial extensor tendon and extensor carpi radialis brevis. Next, through a small longitudinal surgical incision subcutaneous tissue dissection was performed and the two distal screws were implanted using as a landmark, the tubercle of the second metacarpal bone in the dorsolateral position, paying attention to the extensor tendon and the radiodorsal neurovascular bundle on the extensor aspect.

After checking the correct position of the screws, two cover clamps proximal and distal, were closed to secure the screws. Then the wrist module with two rods in neutral position was attached to the two clamps. The wrist module has been designed to be particularly handy, permitting the central joint angulations up to ± 45° in the frontal and sagittal planes.

**Fig 1:**
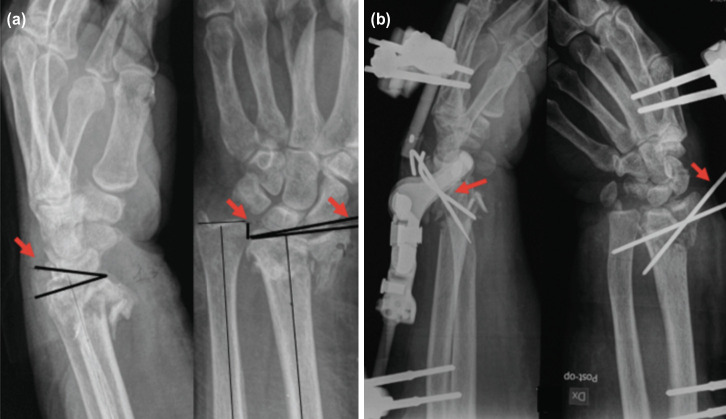
a) Pre-operative radiograph: the arrows showing angles for calculation of pre-operative radiological parameters. (b) Post-operative radiograph: the arrows showing use of K-wires to complement fracture reduction.

**Fig 2:**
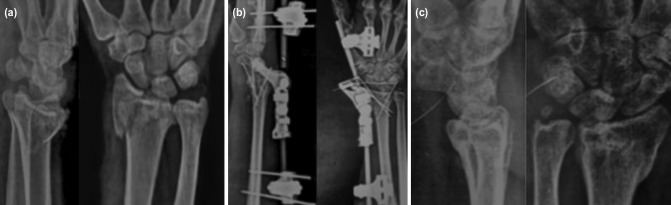
Radiographs of right wrist (a) pre-operative, (b) post-operative, (c) after 12 months.

The insertion of the bone screws and clamps assures that the fracture has been reduced by external manipulation and the EF construct fixed. If there was unacceptable fragment reduction with just the external fixator, K-wire were used additionally to complete the fracture stabilisation (Fig. 1 and 2).

All the patients were instructed in daily pin cleaning. Modular bridging external fixator allows wrist rapid mobilisation as early as the third week by unlocking the wrist module, as shown in surgical technique. The external fixator was removed in the seventh week post-op, the timing of application of these procedures was chosen case-by-case^11^.

A suggestion, range of movement at the wrist is commenced by adjusting the setting at the wrist module. The decision to commence movement in the third post-operative week, supported by satisfactory reduction at surgery, or to remove the external fixator at seven weeks, is determined on a case-to-case basis.

Statistical analysis was performed using STATA/MP 14 for Windows [Stata Corp LP, College Station, USA]. Paired t-test was used to assess variability between baseline and each follow-up. Pearson correlation test was performed. The tests were two-tailed; a p-value of less than 0.05 was considered significant.

## Results

Thirty-five patients (female: 21, male:14; mean age: 62.5), with a mean follow-up of 14.95 months. The main data of the study are summarised in Table I.

**Table I:** Main data of the study

Age	
Mean ±SD	62.5±14.4
Range	27-75
Gender	
Male. n (%)	21 (60%)
Female. n (%)	14 (40%)
BMI (Kg/m2)	
Mean ±SD	27.6±6.4
Side	
Left. n (%)	19 (54.29%)
Right. n (%)	16 (45.7%)
Smoking status	
# of smokers. n (%)	6 (17.14%)
AO Classification	
23-B1. n (%)	-
23-B2. n (%)	3 (8.57%)
23-B3. n (%)	4 (11.43%)
23-C1. n (%)	9 (25.7%)
23-C2. n (%)	12 (34.29)
23-C3. n (%)	7 (20%)
Type of trauma	
High-energy trauma. n (%)	26(74.3%)
Low-energy trauma. n (%)	9 (25.7%)
Surgical time (min)	
Mean ±SD	51±9.7
Range	42-67
Mobilisation timing (days)	
Mean ±SD	24±8.5
Range	14-33
External Fixator Removal Timing	
Mean ±SD	58.4±12.6
Range	46-74

Mean surgical time was 51 minutes (range 42-67). Only in two fractures out of 35 (5.71%), the surgical time was greater than 60 minutes; both fractures were classified as 23-C3, according to AO/OTA classification. 26 fractures out of 35 (74.3%) derived from low-energy trauma, whereas 9 fractures out of 35 were caused by high-energy trauma. Additional k-wires were used in 16 patients out of 35 (45.72%).

Mean mobilisation time was 24 days (range 14-33 days). In all patients, after mobilisation the ROM 0-40° was allowed. Mean external fixator removal time was 58.4 days (range 46-74); Clinical and functional scores over time were summarised in Table II. A significant improvement of the DASH and PRWE scores was observed at time of mobilisation. No significant impairment of these scores were recorded at the following follow-ups (Table II). A significant improvement of the ROM and grip strength was observed at 6 months and 12-months follow up (Table II).

**Table II:** Clinical outcome: differences between each follow-up versus baseline at left side and between each follow-up versus EF Removal at ride side (Paired t-test)

	DASH		PREW		Flexion		Extension		Ulnar radial deviation		Grip strength (kg)	
	Mean	*p*	Mean	*p*	Mean	*p*	Mean	*p*	Mean	*p*	Mean	*p*
Baseline	58.36	-	61.73	-								
EF Mobilisation	48.7	0.035	45.64	0.023								
EF removal	46.65	0.032	42.56	0.022	19.57	-	10.83	-	27.01	-	7.83	-
6 months review	35.43	0.01	38.97	0.002	34.74	0.001	35.71	0.001	45.80	0.001	14.09	0.001
12 months review	15.73	0.001	20.10	0.001	48.44	0.001	56.56	0.001	79.29	0.001	27.83	0.001

EF= External Fixator; f.u.= follow-up

The radiological data are summarised in Table III: a significant improvement of radial inclination, ulnar variance and palmar inclination was observed post-operatively. No significant impairment of these parameters was recorded at the follow-up (Table III). No significant correlations were found between radiological parameters and clinical scores at 12 months follow-up analysing data with Pearson correlation test (Table IV).

**Table III:** Radiological Analysis: differences at each follow-up review versus baseline (Paired t-test)

	Radial inclination (°)		Ulnar variance (mm)		Palmar inclination (°)	
	Mean	*p*	Mean	*p*	Mean	*p*
Baseline	13.5	-	-1.3	-	4.85	-
Post-operatively	21	0.002	1.05	0.02	9.95	0.001
EF Mobilisation	20.5	0.003	1.20	0.01	9.95	0.001
EF removal	20.39	0.003	1.33	0.01	9.83	0.001
12 months review	19.92	0.0035	1.12	0.01	9.76	0.001

EF= External Fixator; f.u.= follow-up

**Table IV:** Correlation between radiological parameters and clinical scores at 12 months follow-up (Pearson Correlation Test)

	DASH		PREW	
	R	*p*	R	*p*
Radial inclination	0.32	0.082	0.35	0.095
Ulnar variance	0.24	0.223	0.27	0.243
Palmar inclination	0.31	0.093	0.33	0.094

Significant correlations were found between AO/OTA classification and additional K-wires analysing data with Pearson correlation test (Table V). The best clinical outcome, in terms of ROM restoration, was observed in type 23-B3 and 23-C1 injuries, although not statistically significant with Pearson correlation test (p>0.05).

**Table V:** Correlation between AO/OTA classification and additional K-wires (Pearson Correlation Test)

AO/OTA classification	Additional k-wires		P
	No	Yes	
23-B2	3	0	0.045
23-B3	4	0	
23-C1	2	7	
23-C2	6	6	
23-C3	4	3	

## Discussion

Distal radius (DRFs) fractures are one of the common injuries observed in orthopaedic clinical practice, with a reported high incidence in young patients involved in high-energy trauma and in post-menopausal women, as a consequence of low-energy trauma in osteoporotic bone^15^. In our study, DRFs were mainly observed in female patients (60%) and low-energy trauma was found to be the main cause of injury (74.3%). High-energy trauma was mainly observed in young male patients, with an age range of 27-42 years old.

Open reduction and internal fixation (ORIF) often permits a stable construct and a good bone healing but results in cosmetically unacceptable incisions^16^. Considering the widespread use of minimally invasive technologies in other orthopaedic surgery^17,18^ their use could be extended to trauma surgery whenever possible. The use of external wrist fixator permits an acceptable fracture healing without exposing the fracture site and compromising the bone vascularisation^19^.

The significant correlations found between AO/OTA classification and additional K-wires analysing data with Pearson correlation test, shows that additional K-wires are useful in the fixation of fracture type 23-C1, 23-C2 where the articular part of radius is not badly fragmented. In these cases, the K-wires were used to fix these fragments, offering the advantage of limiting the distraction forces, thus reducing the effect of ligamentotaxis and improving fracture stability.

After mobilisation, in 9 patients out of 35 a radial inclination mean loss of 2.3° was recorded; in 8 patients out of 35 a palmar inclination mean impairment of 1.5° was observed and in only 2 cases out of 35 an ulnar variance mean reduction of 1.5mm was observed. In none of these patients a revision surgical procedure was needed.

The importance of the restoration of radiological parameters is still a matter of debate^20-23^. According to AAOS guidelines, surgery is recommended in fractures with radial shortening of more than 3mm, a dorsal tilt>10° and an articular step-off more than 2mm. Several studies, however, have shown no significant correlation between radiological parameters and clinical outcome^20-23^. In the current study, no significant correlation was recorded between DASH, PRWE scores and radiological parameters (radial inclination, ulnar variance and palmar inclination).

On the external fixator removal, some limitation of ROM and grip strength was detected, especially in 23-C3 type injuries. At the subsequent follow-ups, however, a significant improvement of ROM and grip strength was recorded. This data is consistent with what is reported by Margaliot *et al* in a systematic review with meta-analysis^24^.

Two complications were recorded in this study. A patient with a 23-C3 injury was found to have a loss of fracture reduction in the first post-operative week, requiring a surgical revision of the external fixator, adding K-wires. Another patient with a 23-B3 injury complained of pain at the fracture site at 60 days post-operatively, and the external fixator removal was delayed by fourteen days.

This study has some limitations. Though the sample size is quite large, the lack of a randomised controlled group is a limitation of this study. The 12-month follow-up is too short to detect any arthritic changes of the wrist. To offset this, it is planned for all the patients to undergo a longer clinical-radiological follow-up.

## Conclusion

This study revealed that the modular external fixator system clinically and radiologically was effective in the treatment of unstable and comminuted distal radial fractures. Additional K-wires were useful addition for fracture fixation when there is unacceptable fragment reduction only with external fixator.

## References

[1] Nellans KW, Kowalski E, Chung KC (2012). The epidemiology of distal radius fractures.. Hand Clin..

[2] Koo OT, Tan MK, Chong KS (2013). Distal radius fractures: an epidemiological review.. Orthop Surg..

[3] De Vitis R, Passiatore M, Cilli V, Maffeis J, Milano G, Taccardo G. (2020). Intramedullary nailing for treatment of forearm non-union: Is it useful? - A case series.. J Orthop..

[4] Brogan DM, Richard MJ, Ruch D, Kakar S (2015). Management of Severely Comminuted Distal Radius Fractures.. J Hand Surg Am..

[5] Myderrizi N (2011). Factors predicting late collapse of distal radius fractures.. Malays Orthop J..

[6] Maccagnano G, Noia G, Vicenti G, Baglioni M, Masciale MR, Cassano GD (2021). Volar locking plate versus external fixation in distal radius fractures: A meta-analysis.. Orthop Rev (Pavia)..

[7] Lichtman DM, Bindra RR, Boyer MI, Putnam MD, Ring D, Slutsky DJ (2010). Treatment of distal radius fractures.. J Am Acad Orthop Surg..

[8] Handoll HH, Madhok R (2003). Surgical interventions for treating distal radial fractures in adults.. Cochrane Database Syst Rev..

[9] Grewal R, MacDermid JC (2007). The risk of adverse outcomes in extra-articular distal radius fractures is increased with malalignment in patients of all ages but mitigated in older patients.. J Hand Surg Am..

[10] Wang D, Shan L, Zhou JL (2018). Locking plate versus external fixation for type C distal radius fractures: A meta-analysis of randomized controlled trials.. Chin J Traumatol..

[11] Orthofix. http://https://www.galaxyfixation.com/wp-content/uploads/2020/06/GF-1101-OPT-E0.pdf.

[12] Capo JT, Swan KG Jr, Tan V. (2006). External fixation techniques for distal radius fractures.. Clin Orthop Relat Res..

[13] Cooney WP (1989). Management of Colles' fractures.. J Hand Surg Br..

[14] Walenkamp MM, Vos LM, Strackee SD, Goslings JC, Schep NW (2015). The Unstable Distal Radius Fracture-How Do We Define It? A Systematic Review.. J Wrist Surg..

[15] Levin LS, Rozell JC, Pulos N (2017). Distal Radius Fractures in the Elderly.. J Am Acad Orthop Surg..

[16] Ricciardi L, Sturiale CL, Pucci R, Reale G, Stifano V, Izzo A (2019). Patient-Oriented Aesthetic Outcome After Lumbar Spine Surgery: A 1-Year Follow-Up Prospective Observational Study Comparing Minimally Invasive and Standard Open Procedures.. World Neurosurg..

[17] Proietti L, Ricciardi L, Noia G, Barone G, Valenzi E, Perna A (2019). Extensive Spinal Epidural Abscesses Resolved with Minimally Invasive Surgery: Two Case Reports and Review of the Recent Literature.. Acta Neurochir Suppl..

[18] Proietti L, Perna A, Schiro GR, Noia G, Fumo C, Tamburrelli FC (2019). Residual mobility after removal of instrumentation in patient, with type a2-a3 vertebral fractures, treated with percutaneous pedicle screw fixation.. J Biol Regul Homeost Agents..

[19] De Vitis R, Passiatore M, Perna A, Tulli A, Pagliei A, Taccardo G. (2019). Modified Matti-Russe technique using a "butterfly bone graft" for treatment of scaphoid non-union.. J Orthop..

[20] Roh YH, Lee BK, Baek JR, Noh JH, Gong HS, Baek GH (2015). A randomized comparison of volar plate and external fixation for intra-articular distal radius fractures.. J Hand Surg Am..

[21] Diaz-Garcia RJ, Oda T, Shauver MJ, Chung KC. (2011). A systematic review of outcomes and complications of treating unstable distal radius fractures in the elderly.. J Hand Surg Am..

[22] Dario P, Matteo G, Carolina C, Marco G, Cristina D, Daniele F (2014). Is it really necessary to restore radial anatomic parameters after distal radius fractures?. Injury..

[23] Cherubino P, Bini A, Marcolli D (2010). Management of distal radius fractures: treatment protocol and functional results.. Injury.

[24] Margaliot Z, Haase SC, Kotsis SV, Kim HM, Chung KC (2005). A meta-analysis of outcomes of external fixation versus plate osteosynthesis for unstable distal radius fractures.. J Hand Surg Am..

